# Nutritional, chemical and microbiological changes during fermentation of tarhana formulated with different flours

**DOI:** 10.1186/s13065-015-0093-4

**Published:** 2015-04-02

**Authors:** Aysegul Kumral

**Affiliations:** Department of Food Engineering, Faculty of Agriculture, Uludag University, 16059 Gorukle, Bursa Turkey

**Keywords:** Whole wheat, Chickpea, Phytic acid, Lactic acid bacteria, Yeasts, Proteins

## Abstract

**Background:**

Tarhana is a fermented cereal product that is used for the preparation of one of the favourite soups in Turkish cuisine. Tarhana is a mixture of wheat flour, yoghurt, baker’s yeast, salt, and various vegetables, spices and seasonings. It is obtained by mixing all ingredients in the recipe and afterwards the mixture is let to ferment at room temperature. Following fermentation tarhana is dried or frozen for long term storage. In this study, to improve the nutritional benefits of tarhana, whole wheat and chickpea flours were used as the sole source of flour. The changes in the phytic acid content, proteins and fermentation products were investigated in addition to some microbiological and chemical characteristics.

**Results:**

The effect of flour type on the phytic acid content was significant. No differences were observed in the glutenin band patterns of the wheat and whole wheat flours and their tarhana samples. Conversely, for the gliadin fractions, the bands of the wheat and whole wheat flours were more intense than their tarhana samples. The changes in the glutelin and prolamin fractions of the chickpea flour and the resultant tarhana dough were similar to the glutenin and gliadin fractions of wheat and whole wheat flours and their tarhana samples. In all samples, the yeasts displayed an undulant growth pattern and the effect of flour type and fermentation time on yeast growth was significant (*P* < 0.01). The effect of flour type (*P* < 0.01) and fermentation time (*P* < 0.05) on mesophilic LAB was significant. Similar behaviours were observed with the mesophilic LAB in all samples and their numbers remained closed to their initial numbers. The growth of thermophilic LAB was not influenced by the flour type, but the effect of fermentation time was significant (*P* < 0.01).

**Conclusions:**

The whole wheat and chickpea tarhana are found to be good alternatives to classical tarhana with their higher nutritional benefits but further investigations are needed for the assessment of their sensory properties.

## Background

Tarhana can be defined as a mixture of wheat flour, yoghurt, baker’s yeast, salt, and various vegetables, spices and seasonings and obtained by drying or freezing the mixture following fermentation. Different ingredients such as legumes are added to the recipe in different regions of Turkey. Tarhana is one of the favourite soups in Turkish cuisine and it is obtained by simmering the thawed mixture or the reconstituted dry powder [1]. Since it is easily digestible and rich in nutrients (vitamins, minerals, proteins and fermentation products like organic acids), it is preferred for the feeding of children, elderly and ailing people [2,3]. Tarhana fermentation lasts for 1–7 days and alcohol and lactic acid fermentations occur concurrently by the activity of microorganisms coming from the ingredients such as baker’s yeast and yoghurt [4]. Similar cereal products with different names (trahanas in Greece, tarhonya in Hungary, talkhuna in Finland, kishk in Syria, Palestine, Jordan, Lebanon and Egypt, kushuk or kushik in Iran and Iraq) are produced and consumed extensively in different countries [5].

Recent scientific research data showed the benefits of whole grain products consumption on the prevention of cancer, obesity and some chronic diseases like coronary heart disease and diabetes mellitus [6]. It is reported that, the prevention was caused by some constituents of whole grains such as dietary fibre, minerals, vitamins, lignans, antioxidants, phenolic compounds, fats and starch. These compounds are widely distributed in the germ and bran of cereals and most of them lost during the refining steps of flour processing [7,8].

The chickpea (*Cicer arietinum* L.) is drawing attention recently, and it is included in some food preparations due to its high protein digestibility and rich protein, carbohydrate, B vitamin and mineral content [9,10]. It is also included in traditional tarhana recipes in addition to the basic ingredients, to improve the nutritional quality.

In this study, whole wheat and chickpea flours were selected as sole source of flour to improve the nutritional value of tarhana. The changes in protein patterns and phytic acid content during tarhana fermentation were monitored in addition to the microbiological and other chemical characteristics. Wheat gluten proteins have technological importance since they are responsible for dough properties such as extensibility, elasticity, hydration and gas retention [11,12], but they also cause celiac disease [13]. Whole grain foods and legumes are also rich in phytate, the salt form of phytic acid (PA). Phytate forms complexes with minerals like Fe^2+^, Zn^2+^ and Ca^2+^ and prevents mineral absorption in the human intestine and causes health problems like mineral deficiency [14,15]. But, on the other hand, it acts as a therapeutic and preventive agent for cancer treatment [16].

## Results and discussion

### Microbiological changes during fermentation

During tarhana production, two types of fermentations (alcohol and lactic acid fermentations) occur concurrently by the activity of microorganisms coming from the ingredients such as baker’s yeast and yoghurt [4]. The yeast (*Saccharomyces cerevisiae*) and lactic acid bacteria generate ethanol, carbon dioxide and lactic acid as well as other fermentation products like aldehydes, ketones and different organic acids. By this way, they give tarhana the typical taste and aroma and contribute to its preservation by lowering the pH [4,17].

**Figure 1:**
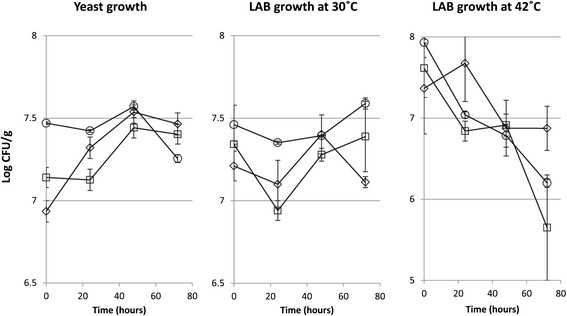
**Changes in microbial populations during tarhana fermentation (◊, wheat flour; □, whole wheat flour; ○, chickpea flour).**

During tarhana fermentation, yeasts displayed an undulant growth pattern in all samples, and their numbers increased during the first two days (Figure 1). The effect of flour type and fermentation time on yeast growth was significant (*P* < 0.01). The increase in yeast number indicated that yeasts fermented the free sugars in the mixture and involved in the tarhana fermentation. As determined in Erbas *et al.* [18], the number of yeasts decreased due to the increase in acidity degree on the third day of fermentation but still remained higher than the initial number of yeasts in wheat and whole wheat tarhana. The highest yeast counts were reached by chickpea tarhana (CT, 48 h), but at the end of fermentation, number of yeasts decreased below the initial number due to the marked increase in the acidity of the sample. The number of lactic acid bacteria (LAB) in the tarhana samples during fermentation was monitored at two different incubation temperatures (30°C for mesophilic and 42°C for thermophilic bacteria, Figure 1). The effect of flour type (*P* < 0.01) and fermentation time (*P* < 0.05) on mesophilic LAB was significant. Similar behaviours were observed with the mesophilic LAB in all samples and their numbers remained closed to their initial numbers. The growth of thermophilic LAB was not influenced by the flour type, but the effect of fermentation time was significant (*P* < 0.01). In whole wheat flour tarhana (WWFT) and CT, a marked decrease in the number of thermophilic LAB was observed. This could be derived from the temperature (30°C) of the tarhana fermentation and the marked increase in acidity of the sample. There was no similarity between the behaviours of mesophilic and thermophilic LAB.

Sengun *et al.* [19] reported the similarity in the number of mesophilic and thermophilic lactobacilli incubated at two different temperatures. Moreover, Daglioglu *et al.* [17] detected maximal LAB counts on the second day of fermentation and reported a stable or declining trend on the following fermentation days. These contradictory results demonstrate that the change in the number of lactic acid bacteria is dependent on the composition of the sample as well as the fermentation time and temperature.

### Acidity degree, organic acids and ethanol contents

The major organic acid in fermented tarhana dough is lactic acid [20]. The total acidity of the samples is calculated as lactic acid (LA, %) because the amount of the other organic acids in tarhana and similar products are in such small concentrations [21]. According to the Tarhana Standard (TS 2282) of the Turkish Standards Institute [22], the acidity degree of tarhana should be between 15 and 40 degrees. The acidity of the samples is displayed as both “acidity degree” and “total acidity” (Table 1) to simplify the comparison. The activity of acidic fermentations can be measured by the acidity formed, and the fermentation process was monitored by the degree of acidification. The length of the fermentation process was defined as the time when the samples reached the upper limit of acidity degree (40), as stated by the Tarhana Standard. The fermentation of the tarhana samples was terminated at the end of 3 days because the acidity degree of all samples surpassed 40, and then, all of the samples were kept frozen until analyses. The acidity degree of the samples ranged between 17–63.6 degrees (1.6-5.7 g/100 g LA). Significant differences were observed at the initial acidity degree measurements of the samples as a result of the different chemical compositions of the flours [23]. Higher initial and final acidity degrees were detected in WWFT and CT than in wheat flour tarhana (WFT), which corroborates the results from Bilgicli and Ibanoglu [21] and Bilgicli *et al.* [24] where the wheat germ/bran addition increased the initial and final acidity degrees of the tarhana samples. The acidity degree/total acidity of all samples increased significantly over time (*P <* 0.01) because of the activity of the fermenting microbiota [18].

**Table 1:** **Moisture, total acidity, phytic acid, organic acids and ethanol contents**

**Flours**	**Time (Hours)**	**Moisture content** ^**1**^ **(%)**	**Acidity degree/Total acidity (LA %)** ^**1, 2**^	**Phytic acid** ^**1, 2**^ **(mg/100 g)**	**L-Lactic acid** ^**1**^ **(g/100 g)**	**D-Lactic acid (g/100 g)**	**Acetic acid** ^**1**^ **(g/100 g)**	**Ethanol** ^**1**^ **(g/100 g)**
**Wheat (WF)**	-	12.5a	-	109.9b	-	-	-	-
**Whole wheat (WWF)**	-	13.1a	-	464.5a	-	-	-	-
**Chickpea (CF)**	-	11.4b	-	493.6a	-	-	-	-
**Tarhana samples**	**Time (Hours)**	**Moisture content** ^**1**^ **(%)**	**Acidity degree/Total acidity (LA %)** ^**1,2**^	**Phytic acid** ^**1,2**^ **(mg/100 g)**	**L-Lactic acid** ^**1**^ **(g/100 g)**	**D-Lactic acid (g/100 g)**	**Acetic acid** ^**1**^ **(g/100 g)**	**Ethanol** ^**1**^ **(g/100 g)**
**Wheat flour** **Tarhana (WFT)**	0	24.9 k	17.0j/1.6j	75.6c	0.41b	0.04	0.17de	1.63 cd
	24	31.4i	39.2 g/3.5 g	70.5c	0.48ab	0.05	0.24cde	2.04bc
	48	35.5d	41.7f/3.8f	67.2c	0.45ab	0.05	0.29abc	2.39ab
	72	33.4 g	40.7 fg/3.7 fg	60.5c	0.42ab	0.04	0.26bcde	2.31ab
**Whole wheat flour tarhana (WWFT)**	0	24.9 k	23.6i/2.2i	374.7b	0.49ab	0.05	0.18de	1.49d
	24	28.2j	53.3d/4.8d	367.2b	0.44ab	0.03	0.23cde	2.00bc
	48	34.6e	58.5c/5.3c	363.3b	0.47ab	0.06	0.27bcd	2.67a
	72	34.2f	61.2b/5.5b	361.9b	0.44ab	0.04	0.34ab	2.61a
**Chickpea flour tarhana (CFT)**	0	31.8 h	35.5 h/3.2 h	411.1a	0.47ab	0.04	0.17e	1.70 cd
	24	36.2c	46.4e/4.2e	407.9a	0.49ab	0.04	0.31abc	2.02bc
	48	38.7a	59.0c/5.3c	404.7a	0.59a	0.04	0.35ab	2.30ab
	72	36.9b	63.6a/5.7a	400.1a	0.59a	0.03	0.36a	2.30ab
**ANOVA**								
**Flour type**		**	**	**	**	NS	**	NS
**Time**		**	**	*	NS	NS	**	**
**Flour type x Time**		**	**	**	*	NS	*	*

^**1**^ Values are means and those in the same column with different letters are significantly different (*P <* 0.01).

^**2**^On dry basis**;** *, Significant at 0.05 level, **, Significant at 0.01 level.

Throughout fermentation, with the activity of lactic acid bacteria and yeasts, lactic acid, ethanol, carbon dioxide and various other organic compounds are produced and provide the characteristic taste and flavour of tarhana [25]. The organic acids in tarhana come from its ingredients (yoghurt, tomato and pepper pastes, etc.) and are produced by the metabolism of fermentable sugars [18]. Lactic acid in fermented tarhana dough comes from yoghurt and produced from the fermentable carbohydrates found in the flour-yogurt mixture by the homo- and hetero-fermentative LAB. In addition to lactic acid, other organic acids, including acetic, propionic and pyruvic acids, are formed by lactic, acetic and propionic acid bacteria found in the flour. A small amount of citric acid is found in the tarhana coming from vegetables used for preparation [18,20]. The organic acid contents of the tarhana samples are shown in Table 1. The effect of flour type and fermentation time on the L-lactic acid and acetic acid contents of tarhana samples was significant (*P <* 0.05). The acetic acid content of the samples increased as fermentation time elapsed. Despite the increase in the total acidity of all samples and the number of lactobacilli and streptococci present during fermentation (over log 6.5 CFU g^−1^), no increase was detected in L-lactic acid content of any sample. The lactic acid produced during fermentation may be used as a substrate by the propionic acid bacteria found in flour, which may explain the previously described result by Erbas *et al.* [18]. During tarhana fermentation, acid formation occurs simultaneously with ethanol production. The ethanol content of the samples is affected by flour type and fermentation time (*P <* 0.05), and increased during fermentation by the activity of yeasts and LAB [25].

### Phytic acid content

PA, is an organic compound occurring in many cereal grains, oilseeds and legumes. The salt form of PA, phytate, is an anhydrous form of phosphate with an affinity for divalent food minerals such as Fe^2+^, Zn^2+^ and Ca^2+^. The resulting phytate-mineral complexes are generally insoluble at physiological pH, which obstructs mineral uptake in the human intestine and leads to mineral deficiencies, such as iron deficiency anaemia [14,15]. Phytic acid hydrolyzes into inositol and phosphates or phosphoric acid by enzymatic activities (phytases) or nonenzymatic breakdown. Fermentation and microorganisms with phytase activity are effective together with several other methods (germination, cooking, soaking, and autolysis) on decreasing the negative effect of phytic acid on mineral absorption [16]. But, on the other hand, phytic acid acts as a therapeutic and preventive agent for cancer treatment by reducing the proliferation of malignant cells and increasing the conversion of them into normal ones [26]. The tarhana dough samples were analysed for the detection of the effect of flour type and fermentation process on the PA content. The PA content of the flours and the tarhana samples are presented in Table 1. The effect of flour type on the PA content was significant (*P <* 0.01), and the PA content of the tarhana samples was lower than the corresponding flours. The PA contents of wheat flour (WF) and its tarhana were lower than whole wheat flour (WWF) and chickpea flour (CF) and their respective tarhana samples, which corroborates the results from Požrl *et al.* [27]. Bilgicli and Ibanoglu [21] and Kose and Cagindi [23] reported a reduction in the PA content of tarhana samples during the entire fermentation process. In contrast to these previous results, no significant decrease was detected in the PA content of the tarhana samples. The reduction of PA in tarhana is attributed to the inherent phytase activity in the fermenting mixture [21], but it is also influenced by the initial PA content, the degree of flour extraction, the fermentation time and the presence of calcium salts as well as the temperature, dough acidity, pH, yeast type and enzymes added to dough [27,23]. PA level of the tarhana samples were not changed during the whole fermentation time, so it is not affected from none of factors such as the phytase activity of the flours, LAB (from yoghurt) and yeasts, (from Baker’s yeast) and changes in the acidity. Comparatively lower PA levels observed in doughs than their respective flours, because of the rate of flour in the total tarhana mix obtained by the addition of all other ingredients.

### Changes in electrophoretic characteristics of proteins during fermentation

Wheat proteins are classified as gluten proteins or non-gluten proteins. The non-gluten proteins are albumins and globulins. They are easily extractable in salt solutions and their molecular weights (MWs) are lower than 25000 Da [12]. Wheat gluten proteins are gliadins and glutenins, which are grouped based on their extractability (gliadins) or unextractability (glutenin) in aqueous alcohols. The gliadins (MW ranging from 30000 to 80000 Da) are soluble in aqueous alcohols, while both gliadins and glutenins (MW ranging from 80,000 Da to several million Da) are soluble in dilute acetic acid. Gluten proteins are chiefly responsible for dough properties such as extensibility and elasticity and they contribute to the dough hydration and gas retention [5,11,12]. The strength and elastic properties are mainly imparted by the glutenin fraction, while the gliadin fraction can play a role in determining dough extensibility [28]. However, glutens also cause celiac disease, which is a disorder associated with impaired intestinal digestive function that occurs under the influence of the gluten proteins found in food [13]. The gluten proteins are affected by the metabolic activities of bacteria and cereal enzymes and degradation and depolymerisation of proteins are observed during fermentation [11].

**Figure 2:**
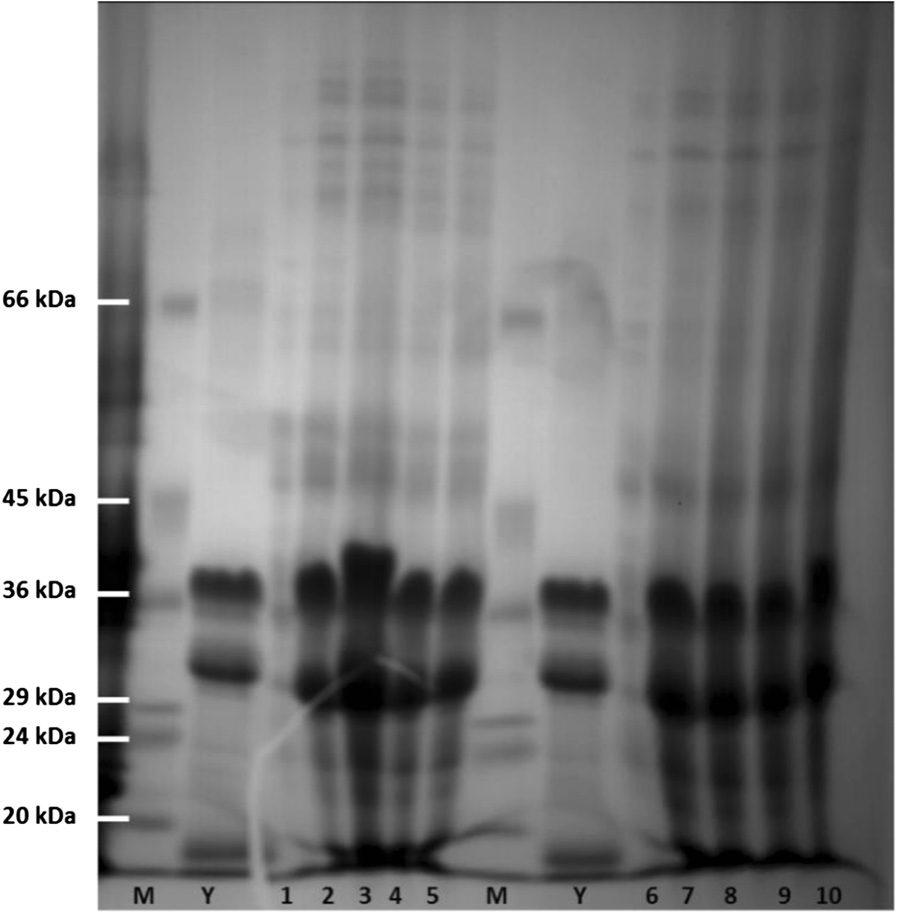
**SDS-PAGE patterns of the samples M: Marker, Y: Yoghurt, 1: glutenin fractions of WF, 2: WFT day 0, 3: WFT day 1, 4: WFT day 2, 5: WFT day 3, 6: WWF, 7: WWFT day 0, 8: WWFT day 1, 9: WWFT day 2, 10: WWFT day 3.**

**Figure 3:**
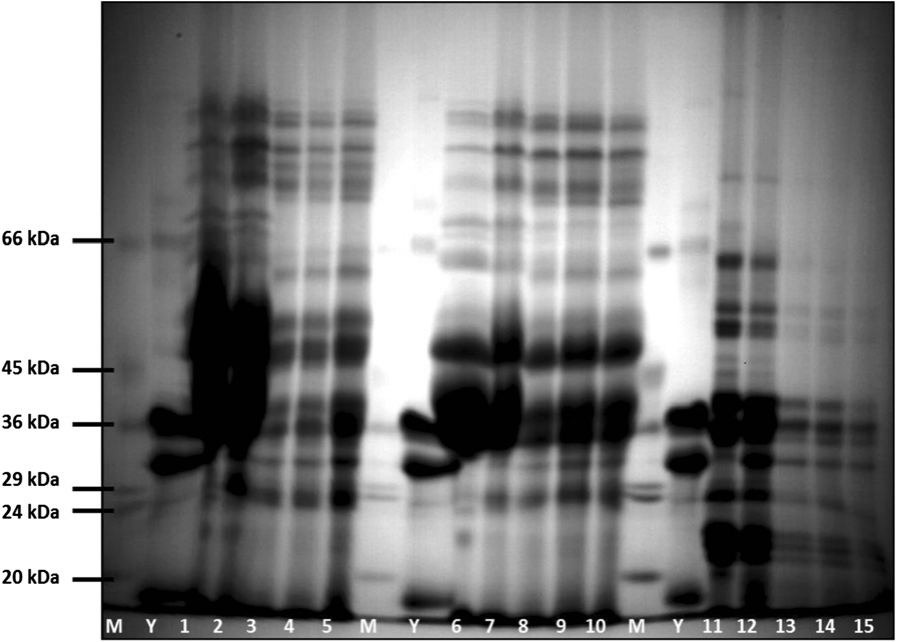
**SDS-PAGE patterns of the gliadin and prolamin fractions.** M: Marker, Y: Yoghurt, 1: WF, 2: WFT day 0, 3: WFT day 1, 4: WFT day 2, 5: WFT day 3, 6: WWF, 7: WWFT day 0, 8: WWFT day 1, 9: WWFT day 2, 10: WWFT day 3, 11: CF, 12:CT day 0, 13: CT day 1, 14: CT day 2, 15: CT day 3.

**Figure 4:**
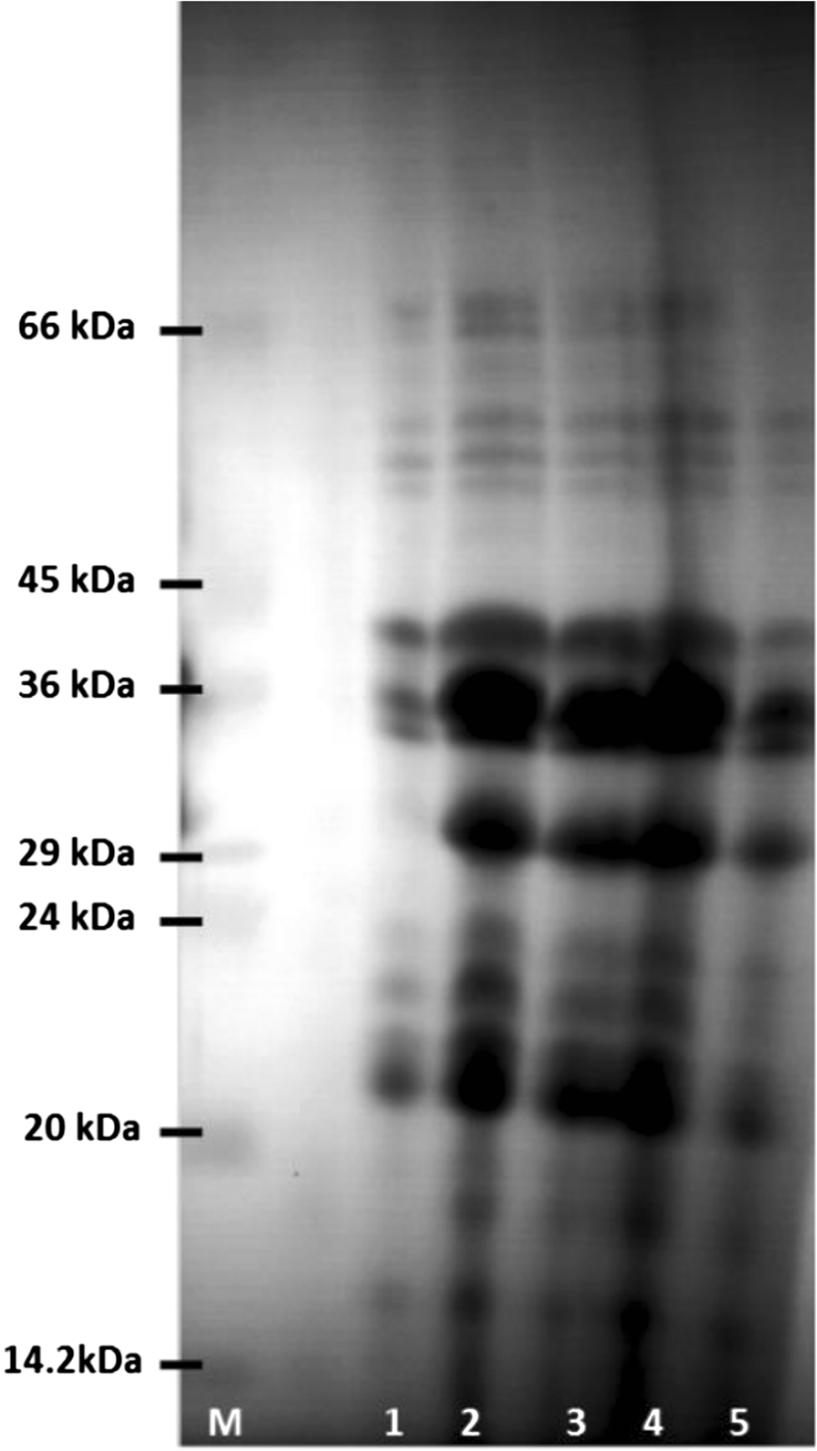
**SDS-PAGE patterns of the glutelin fractions of the chickpea samples extracted with the glutenin extraction procedure.** M: Marker, 1: CF, 2:CT day 0, 3: CT day 1, 4: CT day 2, 5: CT day 3.

The changes in the electrophoretic patterns of the flours and the tarhana dough samples are demonstrated in Figures 2, 3 and 4. No differences were observed in the glutenin band patterns (relative band mobilities) of wheat and whole wheat flours and their respective tarhana dough samples (Figure 2). After the tarhana dough was obtained by mixing the flour with yoghurt and other ingredients (day 0), the band intensities increased, but no changes were observed in the band patterns and band intensities during the subsequent fermentation days (Figure 2). Similar results were obtained by Di Cagno *et al.* [29], who determined that glutenins were not hydrolysed by LAB activity during sourdough fermentation. However, in contrast, the gliadin band intensities of the wheat and whole wheat flours were more intense than their respective tarhana dough samples (Figure 3). The gliadin band intensities became less intense during the subsequent fermentation days (Figure 3) because of protein hydrolysis by the proteolytic enzyme activities of the fermenting microbiota [30].

The storage proteins of the chickpea have been fractionated into salt soluble (globulin), water soluble (albumin), alcohol soluble (prolamin) and acid/alkali soluble (glutelin) fractions as well as the other remaining proteins [31]. The glutelin and prolamin fractions of the chickpea were extracted with the glutenin and gliadin extraction procedures, and their electrophoretic patterns are presented in Figures 3 and 4. The relative glutelin band mobilities of the chickpea flour and its respective tarhana dough were similar (Figure 4). The glutelin band intensities of chickpea flour were less intense than its tarhana dough, which was previously demonstrated by WF and WWF (Figure 4). Similarly, the bands became more intense after mixing with yoghurt and the other tarhana ingredients. However, in contrast with the WFT and WWFT doughs, a marked decrease in the band intensities was observed on the third day of CT fermentation from the hydrolysis of the glutelins, which was predominantly dependent on acidity [32]. The results for the prolamin fractions of CF and CT were similar to the gliadin fraction of WF and WWF and their resultant tarhana doughs.

## Experimental

### Materials

The wheat flour, whole wheat flour and chickpea grains were obtained from local markets in Bursa (Turkey). The chickpea flour was obtained by milling chickpea grains with a grinder (Deima Electro-mechanic Products, Istanbul, Turkey) after thawing.

### Fermentation process

In tarhana recipes, 500 g of yogurt, 100 g of onions, 50 g of tomato paste, 75 g of red pepper paste, 10 g of baker’s yeast and 75 g of salt were used per 1000 g of each flour type. To obtain the tarhana dough, all ingredients in the recipe were mixed and kneaded. The resulting dough samples were fermented for 72 hours in an incubator at 30°C. Samples were taken daily to monitor the fermentation process. All experiments were carried out in triplicate. The fermented dough samples were kept in the freezer for further storage.

### Microbiological assays

Tarhana samples were taken every 24 h during fermentation. The samples were transferred aseptically into 0.1% peptone water and the dilutions were mixed or spread on different media and incubated under the appropriate conditions. For mesophilic and thermophilic lactic acid bacteria, de Man-Rogosa-Sharpe agar (Merck, KGaA, Darmstadt, Germany) containing 0.02% sodium azide was incubated for 48 h at 30°C and 42°C, respectively and for yeasts and moulds, Rose Bengal Chloramphenicol Agar (Oxoid, England) containing supplements (Oxoid, England) was incubated at 30°C for 48 h [19].

### Acidity degree, organic acids and ethanol contents

The acidity degree of the tarhana samples was measured according to the Tarhana Standard (TS 2282) of Turkish Standards Institute [22]. In the standard, the acidity degree of tarhana was described as the volume of 1 N NaOH solution consumed to neutralise the free acids in 100 g of tarhana. To evaluate the acidity degree, 50 mL of 67% neutralised ethyl alcohol was added to ten g of the sample, this solution was shaken in a closed container for five min, and then it was filtered. Next, ten mL of the filtered solution was removed; and two to three drops of phenolphthalein indicator were added to the ten mL portion, which was titrated with 0.1 N NaOH.

The organic acids (D/L-lactic acid and acetic acid) and ethanol contents of the samples were analysed spectrophotometrically using enzymatic analysis kits from Boehringer Mannheim according to the manufacturer instructions (R-Biopharm AG, Darmstadt, Germany).

### Phytic acid content

The PA content of the tarhana samples was measured according to Haug and Lantzsch [33]. In brief, the samples were extracted with 0.2 N HCl and then 0.5 mL of the extract were mixed with a ferric solution (0.2 g FeNH_4_(SO_4_)_2_ · 12H_2_O dissolved in 100 mL of 2 N HCl and increased to 1000 mL with distilled water) in a well-stoppered glass tube. The contents of the tubes were heated in a boiling water bath for 30 minutes. After cooling in an ice bath, 2 mL of the 2,2′-bipyridine solution (10 g 2,2′-bipyridine and 10 mL thioglycollic acid were dissolved in distilled water and then increased to 1000 mL) was added to the test tube and mixed. The absorbance of the mixture was evaluated immediately against distilled water at 519 nm using a UV/Vis spectrophotometer (Biotek Instruments, Inc. USA).

### Electrophoretic characteristics of proteins

The albumin/globulin, prolamin (gliadin for wheat samples) and glutelin (glutenin for wheat samples) protein fractions of the tarhana and yoghurt were extracted according to Bertrand-Harb *et al.* [34]. The pellets were obtained after all of the extractions were dissolved in a buffer containing 0.0625 M Tris–HCl, 2% SDS, 10% glycerol and 1.5% dithiotreitol (DTT). Each protein fraction was subjected to SDS-PAGE at 25 mA for 3 hours at 24°C, according to Laemmli [35]. The acrylamide concentrations of the stacking and separating gels were 4% and 12%, respectively.

### Statistical analysis

The data were subjected to variance analysis using JMP 7.0 (SAS Institute Inc., Cary, NC, USA) for Windows. The differences between means were determined by Tukey’s test at *P <* 0.01 and 0.05.

## Conclusion

In the present study, results of the microbiological analyses showed that the yeasts were involved in the tarhana fermentation even if they were affected by the increased acidity of the samples. It is also detected that, there was no similarity between the behaviours of mesophilic and thermophilic LAB and the growth of LAB was affected by the availability of the fermentable carbon and nitrogen sources of the sample, acidity degree, fermentation time and temperature.

The PA content of tarhana samples were influenced by the type of flour used. PA content of the tarhana samples was lower than the corresponding flours, but no significant change was detected in the PA level during fermentation. The chickpea and whole wheat tarhana, with their higher PA content, are found to be promising alternatives to wheat tarhana for cancer prevention.

No significant differences were observed in the glutenin relative band mobilities of wheat and whole wheat flours and their respective tarhana dough samples. The glutenin band intensities increased after tarhana dough obtained but, no significant changes were observed in the band patterns and band intensities during the subsequent fermentation days. In contrast with the glutenin fractions, the gliadin band intensities of the wheat and whole wheat flours became less intense following dough mixing and during the subsequent fermentation days. The changes in the glutelin fraction of CT were similar with the glutenin fractions of WFT and WWFT but, distinctively a marked decrease in the band intensities was observed on the third day of CT fermentation. The results for the prolamin fractions of CF and CT were similar to the gliadin fraction of WF and WWF and their resultant tarhana doughs.

The whole wheat and chickpea tarhana are good alternatives to classical tarhana with their higher nutritional benefits but the different taste and odour of chickpea tarhana and bran in whole wheat tarhana are the handicaps of these products. Further investigations are needed for the amendment of their sensory properties.

## Abbreviations

LAB: Lactic acid bacteria
CHD: Coronary heart diseases
PA: Phytic acid
CT: Chickpea tarhana
WFT: Wheat flour tarhana
WWFT: Whole wheat flour tarhana
LA: Lactic acid
WF: Wheat flour
WWF: Whole wheat flour
CF: Chickpea flour
MWs: Molecular weights
